# Integrating formative and summative feedback in online situational judgement tests: effects of feedback design on medical students’ motivational and cognitive learning factors

**DOI:** 10.1080/10872981.2026.2639198

**Published:** 2026-03-02

**Authors:** Sabine Reiser, Kristina Schick, Sylvia Irene Donata Pittroff, Laura Janssen, Pascal O. Berberat, Martin Gartmeier, Johannes Bauer

**Affiliations:** a Faculty of Education, University of Erfurt, Erfurt, Germany; b Institute for Planetary Health Behaviour, University of Erfurt, Erfurt, Germany; c TUM Medical Education Center, Department Clinical Medicine, Technical University of Munich, Munich, Germany; d Institute of Medical Education, Medical Faculty and University Hospital Carl Gustav Carus, TUD Dresden University of Technology, Dresden, Germany

**Keywords:** Situational judgement test, formative and summative assessment, task-based feedback, performance-based feedback, medical communication competence, medical education

## Abstract

**Background:**

Assessment plays an important role in teaching and learning medical communication. Formative assessment is increasingly recognised as a crucial tool for improving learning outcomes and supporting competence development. In this study, we developed and evaluated different versions of automated formative and summative feedback in an online situational judgement test of basic medical communication competence.

**Methods:**

We developed four versions of task-based (formative) feedback, differing in their enrichment strategies (i.e. reflection prompts or expert explanations) and the timing of the feedback (i.e. immediate feedback or post-test feedback), and one performance-based (summative) version featuring a test-score profile. In a randomised controlled trial with *N* = 269 medical students, we evaluated the effects of feedback design on participants’ (i) motivation and (ii) cognitive factors (i.e. cognitive load), as well as on their (iii) feedback perception (e.g. fairness and usefulness of the feedback) and (iv) perceived benefits of the feedback, all of which influence learning. We tested the hypotheses that task-based feedback would be more effective than performance-based feedback (*H*_1_) and that task-based versions would have differential effects on the outcome variables (*H*_2_).

**Results:**

Planned contrast analysis revealed that task-based feedback was not consistently more effective than performance-based feedback across all outcomes (*H*_1_). In line with *H*_2_, analyses of variance revealed differential effects of feedback enrichment and timing: expert explanations enhanced cognitive understanding and perceived feedback benefits, whereas post-test feedback improved motivational outcomes such as perceived competence and fairness.

**Conclusions:**

The results highlight the complexity of determining an optimal feedback approach, as different implementations can have differential effects on the motivational and cognitive factors that further shape learning processes. The findings suggest that an optimal feedback approach depends on specific learning outcomes and student characteristics, highlighting the importance of careful selection of feedback strategies tailored to specific educational goals and contexts.

## Introduction

Teaching and learning medical communication are essential parts of medical education [1–3], and assessing communication competence is crucial in this context [4,5]. Formative assessments embedded in the learning process help educators gauge students’ knowledge and skills and influence learning by guiding study efforts [6,7]. Because their effectiveness depends heavily on the quality of feedback—a major factor in supporting learning [8–10]—there have been increasing calls to integrate feedback into assessments [7,11–13]. In this context, (computer-based) situational judgement tests (SJTs) are increasingly used to assess communicative and social competences (e.g. [4,14–16]). Although SJTs are usually designed for summative purposes, they also hold considerable potential for formative use when feedback is carefully incorporated to encourage ongoing learning [17]. However, few SJTs in medical education integrate feedback, and empirical evidence on effective design principles remains scarce [18,19]. Consequently, there is a significant research gap regarding the design and implementation of feedback within SJTs to enhance learning.

This study presents the development and experimental evaluation of different approaches to integrating automated feedback in an online SJT assessing basic medical communication competence (the video-based assessment of medical communication competence; VA-MeCo [4]). We designed four task-based feedback versions which, based on prior research [8,10,20], are expected to supporting learning. These versions differed with regard to feedback enrichment (expert explanations vs. reflection prompts) and feedback timing (immediate vs. summarised after test completion). In a randomised controlled trial (RCT) with medical students, we compared the effects of these feedback versions with each other and with a control condition providing summative, performance-based feedback, which is frequently considered less effective for supporting ongoing learning [13,21].

### Using SJTs with integrated feedback for formative assessment in medical education

Assessment serves a dual purpose: it enables evaluation of students’ knowledge and skills while also shaping learning by identifying strengths and weaknesses and guiding future study efforts [7,14,22]. In higher education, there is growing demand to integrate feedback into assessment [12,13,22] because of its central role in the learning process [23,24]. Formative assessment^1^, which combines assessment and feedback, is increasingly used in medical education [25–27] and supports competence development by allowing learners and educators to adapt learning and instruction based on assessment results [28]. Substantial evidence demonstrates the positive effects of formative assessment on learning in higher education [29–31].

SJTs are increasingly used in for assessment in medical education [14,15,18,30]. They present examinees with standardised hypothetical scenarios and require judgements or decisions regarding appropriate responses [31,32]. Beyond their good psychometric properties [31,33,34], which have been demonstrated in medical education contexts [14,15,18,30], SJTs offer practical advantages, including efficient online administration for large cohorts and high acceptance among participants [4,15,32]. Delivered online, SJTs allow timely, automated, and potentially personalised feedback [19,35,36], making them well suited for formative purposes. Nevertheless, despite the recognised importance of feedback, little research has investigated its implementation in SJTs [14,37,38].

### Feedback design for enhancing learning

Although feedback can be one of the most influential factors in student learning [9,10,23], its effects on learners can vary greatly depending on its implementation [10,35]. Key design elements include the distinction between task-based and performance-based feedback, the application of enrichment strategies, and feedback timing.

Effective feedback provides information about the gap between learners’ current and target performance and offers guidance on how to close this gap through targeted learning activities [20,36,39]. Accordingly, feedback should be specific and task-focused, providing suggestions for improvement rather than merely indicating correctness or focusing on the learner as a person [36,40,41]. For this purpose, enrichment strategies include providing *expert explanations* of correct answers or *prompting learners’ reflection* on their solution strategies in comparison with the correct response [10]. Expert explanations can function as cognitive scaffolds for learners [42], whereas reflection prompts activate metacognitive processes that support learners in regulating their learning [43]. Reflection encourages the identification of weaknesses, which is particularly effective when learners believe they can improve, fostering positive emotions and perseverance [8]. In contrast, performance-based feedback (e.g. scores or peer comparisons), which remains common in educational practice, is considered less conducive to learning [44,45]. Although such feedback can identify areas warranting improvement, it typically provides only superficial information, does not guide self-regulation or strategy development [10,46], and may even demotivate learners [8].

Another key design element is feedback timing [20,47]. In SJTs, feedback can be provided immediately after each task or after test completion, which may differentially influence subsequent learning through cognitive and motivational mechanisms. Immediate feedback enables just-in-time adjustments by providing task-specific information when it is most salient and may enhance motivation to improve in subsequent tasks [48]. Delayed feedback permits uninterrupted task engagement and can reduce cognitive load (i.e. the mental effort required to process and store information in working memory), which can affect learning and performance [49]. It may also support a more comprehensive reflection on overall performance, as all relevant information is available at once. As empirical evidence regarding these effects in SJTs remains limited, the present study examines their potential effects in an exploratory manner.

Beyond these design elements, learners’ perceptions influence how they engage with feedback. Feedback should be perceived as useful [50] and fair [51], as these perceptions are associated with increased interest and learning [52,53]. From a self-determination theory perspective, fostering motivation requires supporting learners’ sense of competence [54]. Well-designed feedback can address these needs by highlighting strengths, identifying areas for improvement, and offering concrete guidance [55], while also supporting positive affect rather than discouragement [56].

### Aims

Despite the growing use of SJTs in medical education, there is a lack of systematic investigations comparing different feedback strategies in SJTs for formative assessment, including variations in enrichment strategies and timing. Given the critical role of feedback in fostering learning, this study aimed to address this research gap by examining the differential effects of various approaches to integrating automated feedback into an SJT designed to assess medical communication competence (i.e. the VA-MeCo^2^[4]).

Building on the research presented above, we implemented four feedback versions that combine two *enrichment strategies*—providing *reflection prompts* or *expert explanations*—with two different *timings of feedback delivery* (i.e. *after each task* during test processing or combined *after test* processing). To better gauge the effects of these feedback versions, we included performance-based feedback as a fifth condition.

We adopted a multicriteria approach to investigate the effects of the implemented feedback versions, recognising that feedback influences further learning through various motivational and cognitive variables. Specifically, we evaluated the feedback’s impact on four sets of (a) motivational and (b) cognitive outcomes as well as (c) participants’ feedback perception and (d) experienced benefits from feedback, all of which are central to shaping students’ further learning processes.

Our overall research question focused on the effects of these five feedback versions on the mentioned outcome variables. Specifically, we tested the following hypotheses:

*H*_1_: Providing any form of task-based feedback is more effective than providing performance-based feedback across all investigated outcomes.
*H*_2_: Among the four task-based feedback versions, there are differential effects on the four sets of outcomes.

Note that *H*_1_ expresses a literature-based expectation of an average tendency in favour of task-based over performance-based feedback with respect to the investigated dependent variables, rather than an assumption of its deterministic or uniform superiority. *H*_2_ implies that different enrichment and timing strategies may support learning in various ways. We did not anticipate that a single feedback version would optimally foster all outcomes simultaneously. Instead, it is likely that some combinations may be particularly effective in enhancing students’ motivation for further learning, while being less effective in other dimensions. Due to the lack of sufficient evidence in existing research [19,57], we did not specify any hypotheses about the directions of these effects.

## Methods

The VA-MeCo is an SJT designed to assess medical communication competence on three dimensions: (a) advancing the content of the conversation, (b) providing structure, and (c) building relationship with the patient [4]. It consists of 117 items nested within eleven task scenarios, each focusing on a critical aspect or step of patient-physician communication. On average, the test takes approximately 40 minutes to complete. Participants’ test scores are calculated using *raw consensus scoring* [58], one of the most commonly used scoring methods for SJTs. This approach is based on inverted squared deviations of participants’ responses from the expert scoring key. Previous studies have demonstrated the VA-MeCo’s reliability and validity [4,59].

The VA-MeCo feedback versions were developed in a multi-step *construction phase*, involving theoretical and technical development steps as well as cognitive interviews and expert validation. The subsequent *evaluation phase* investigated the abovementioned hypotheses in an RCT. In the following sections, we elaborate on the construction and evaluation phases. Table 1 provides an overview of the studies.

**Table 1.** Participant characteristics of the three conducted examinations.

Data collection	Participants	Sample characteristics
Expert validation of explanations	*n* = 5 SME	*Expertise criteria*: professional experience in medical communication
		*Professional background*: 4 psychologist/other, 1 physician
		*Gender distribution*: 3 females, 2 males
Cognitive Interviews	*n* = 4 SME	*Expertise criteria*: professional experience in medical communication
		*Professional background*: 2 psychologists, 1 psychiatrist, 1 other
		*Gender distribution*: 3 females, 1 male
	*n* = 16 MS	*Semester of study: M* = 10.94 (*SD* = 2.68)
		*Gender distribution:* 12 females, 4 males
Feedback study	*n* = 269 MS	*Semester of study*: *M* = 6.75 (*SD* = 3.79)
		*Gender distribution*: 203 females, 65 males, 1 diverse

Note: SME = subject matter experts; MS = medical students.

### Construction: development strategy and technical implementation of feedback

Drawing on the research discussed above, we developed five different feedback versions (four task-based and one performance-based version): *Immediate Reflection* (IR), *Immediate Explanation* (IE), *Post-Test Reflection* (PR), *Post-Test Explanation* (PE) and *Summative Test Profile* (TP). Table 2 presents an overview of their characteristics. Technical implementation of the feedback features was realised in the open-source learning management system *Moodle* [60], which is frequently used at German universities. This setting ensured broad transferability to interested medical education departments. The design and programming of the feedback features followed a user-centred approach, with a focus on high usability and acceptance by both medical students and faculty.

**Table 2.** Description of the five implemented feedback versions.

Task-based feedback	
*Immediate Reflection (IR)* Graphical visualisation of the difference between the students’ answers and the expert solution Reflection prompts During test processing for each answer option	*Post-Test Reflection (PR)* Graphical visualisation of the difference between the students’ answers and the expert solution Reflection prompts After test processing for each answer option
*Immediate Explanation (IE)* Graphical visualisation of the difference between the students’ answers and the expert solution Expert explanations During test processing for each answer option	*Post-Test Explanation (PE)* Graphical visualisation of the difference between the students’ answers and the expert solution Expert explanations After test processing for each answer option


Performance-based feedback	
*Summative Test Profile (TP, Post-Test only)* Test-score profile: Automatically generated sub-test scores Social comparison with other test takers	

**Task-Based Feedback Versions**. All four task-based versions start with a graphical visualisation comparing students’ answers to the expert solution for each test item, indicating correctness. They differ in enrichment strategies and timing.

In the IR and PR versions, participants receive *reflection prompts* to analyse discrepancies between their responses and the expert solution (for incorrect answers only). They provide open-ended reflections (no word limit), engaging in metacognitive processes to evaluate their reasoning and how it differs from that of the experts. IR provides feedback immediately after each item, while PR provides all feedback right after test completion for broader review.

In the IE and PE versions, the correctness feedback is enriched with *expert explanations* (~ 350 words) outlining the rationale behind the correct solution. These texts, explicitly attributed to experts in medical communication, are based on standards of medical communication (e.g. Calgary-Cambridge Guide [61]), explain criteria for good communication practice and provide best practice examples. All explanations were validated (e.g. regarding medical correctness and comprehensibility) by five subject matter experts in medical communication (see Table 1). Regarding timing, IE provides feedback immediately, whereas PE presents it after test completion.

Performance-Based Feedback Version. In the performance-based feedback version, students receive an individual test-score profile (TP) after completing the test. This includes sum scores for the VA-MeCo’s sub-scales and task-level scores, along with a social comparison with other test takers.

**Cognitive Interviews**. For an initial evaluation and refinement of the developed feedback versions, cognitive interviews with subject matter experts and medical students were conducted (sample description see Table 1) during the construction phase. The participants worked independently on all five feedback versions. Afterwards, they were interviewed on their perception of the feedback features’ usability (e.g. implementation and technical problems) and the learning support provided (e.g. provision of relevant information, support of content-related interest and learning motivation). The results on usability indicated that the participants perceived the handling of all feedback versions as clear and easy to comprehend. Regarding learning support, the participants found the feedback to be cognitively and motivationally engaging.

### Evaluation

**Participants and Design**. Students from all stages of medical education were recruited from the medical education departments of four universities. An a priori power analysis determined that a minimum sample size of *N* = 200 was required to detect medium-sized effects with a statistical power of .80 and a significance level of *α* = .05 in a between-subjects analysis of variance (ANOVA). To account for dropouts and exclusions, we aimed for approximately 30% oversampling above this minimum. Only participants’ who provided informed consent and completed the entire study were included in the analysis, resulting in a final sample of *N* = 269 medical students (age: *M* = 23.34, *SD* = 3.24; gender: 75% female; semester of study: *M* = 6.75, *SD* = 3.79; stage of study: *n* = 109 pre-clinical phase, *n* = 136 clinical phase, *n* = 23 final clinical year). Female students were overrepresented in the sample, which reflects official statistics for medical students in Germany (approximately 65–68% in 2024, depending on medical specialty [62]).

The study employed a between-subjects experimental design, with participants randomly assigned to one of five feedback groups (*n*_*IR*_ = 49, *n*_*IE*_ = 57, *n*_*PR*_ = 53, *n*_*PE*_ = 56, and *n*_*TP*_ = 54). We used the *simple randomisation* procedure [63], assigning individual participants to groups using Microsoft Excel, with equal probability (*p* = .20) for each group. The five groups differed only in the type of feedback they received. The participants were informed in advance about the purpose and content of the study, and they participated voluntarily. We offered monetary incentives (15 EUR per student), which were in line with ethical guidelines and the standard remuneration for student assistants. The study was approved by the Medical Ethics Commission of the Technical University of Munich [2022-244-S-KH].

As outlined above, we investigated the effects on four sets of dependent variables that were categorised in (a) *basic motivational needs* (i.e. interest and perceived competence), (b) *cognitive load* (i.e. intrinsic cognitive load, germane cognitive load and extraneous cognitive load), (c) *perceptions of feedback* (i.e. fairness, usefulness, acceptance and positive affect) and (d) *experienced benefits from the feedback* (i.e. perceived utility for medical communication, and willingness to improve). The variables in (a) and (b) focus on the fundamental characteristics of feedback, while (c) and (d) focus on aspects of its practical application. Additionally, we assessed aspects of usability to identify potential issues related to the user experience.

**Procedure.** All participants completed the experiment in a single session. They first went through a thorough introduction to the study, including screencast videos that explained the handling of the test and the feedback features. Next, the participants completed a shortened version of the VA-MeCo including 14 items (identical across conditions) associated with three video-based patient scenarios. They were selected to represent the three aforementioned dimensions of medical communication competence. As the study focused on the effects of feedback design rather than test performance, a shortened test version was used to keep participant workload manageable, given the additional study tasks and questionnaires. Using the full VA-MeCo (approximately 40 minutes to complete the test tasks, excluding feedback processing) would have increased the risk of fatigue and demotivation without adding value to the study’s objectives.

Depending on their assigned condition, the participants worked on the feedback either during the test (IR and IE) or afterwards (PR, PE and TP). Subsequently, they completed an online questionnaire on the dependent variables, administered within Moodle. Apart from the introductory screencast explaining the experimental tasks, participants in the two reflection-based feedback conditions (IR and PR) did not receive prior reflection training, nor was a specific reflection model applied. As the experiment aimed to isolate the effects of different feedback types and enrichment strategies, it was essential to keep all design elements equivalent across experimental conditions. Providing reflection training exclusively in the reflection-based conditions would have introduced an additional instructional component beyond the feedback manipulation itself and thus constituted a confound, potentially compromising the study’s internal validity.

**Measures.** For all outcome variables, we carefully selected established scales with available evidence supporting their reliability and validity, ensuring they conceptually aligned with our research objectives—some in adapted form to ensure consistency with the study. Table 3 provides an overview, including the reliability estimates from the present study. Further details of the psychometric properties of these scales can be found in the original publications. To assess *basic motivational needs*, interest and perceived competence were measured using items adopted from Wilde et al. [64]. C*ognitive load* was measured using three scales on intrinsic, germane, and extraneous cognitive load [65]. Intrinsic cognitive load refers to the inherent difficulty of the learning material, and germane cognitive load refers to the demands required for elaboration and is conducive to productive learning; in contrast, extraneous cognitive load results from suboptimal design of learning material and is detrimental to learning [65]. Fairness, usefulness, acceptance, and positive affect were measured as aspects of *perceptions of feedback*, using items adapted from Strijbos et al. [66]. Finally, regarding *benefits from the feedback*, perceived utility for medical communication and willingness to improve were assessed [66]. All measures had a 6-point rating scale as the answer format, with higher values indicating a higher prevalence. A codebook with a complete description of all measures, including item texts, is available as open materials [67].

**Table 3.** Internal consistency reliability of the outcome variables.

Measures	N	# Items	α
*Basic motivational needs*			
Interest	267	3	.84
Perceived competence	266	2	.79
*Cognitive load*			
Intrinsic cognitive load	268	2	.77
Germane cognitive load	267	3	.66
Extraneous cognitive load	267	3	.78
*Perceptions of feedback*			
Fairness	268	3	.84
Usefulness	268	3	.89
Acceptance	268	3	.82
Positive affect	269	6	.82
*Benefits from feedback*			
Perceived utility for medical communication	269	3	.87
Willingness to improve	268	2	.53

Note: *α* = Cronbach’s *α* for the (sub-)scales used in the feedback study.

**Statistical Analyses.** We tested *H*_1_ using planned contrast analysis [68,69], which is preferable to traditional ANOVA for testing specific patterns of expected mean differences among multiple groups. Since *H*_1_ predicted that each group receiving task-based feedback (i.e. IR, IE, PR and PE) would outperform the group receiving performance-based feedback (i.e. TP) on the respective dependent variables, we used *simple last contrasts*[70]. Accordingly, four contrasts were calculated, each comparing one task-based feedback condition to the performance-based feedback condition, which served as the reference group (see supplementary Table S1). A significance level of *p* ≤ 0.05 was applied to all analyses, with two-tailed testing. To correct for multiple testing, we applied the Benjamini–Hochberg procedure [71]. Effect sizes for planned contrasts were assessed using Pearson’s *r*, with values of 0.10, 0.30, and 0.50 representing small, medium and large effects, respectively [72].

Since we had no directional hypotheses about the differences among the four groups receiving task-based feedback (*H*_2_), we employed standard multivariate ANOVA (MANOVA) to test the effects on all outcome variables simultaneously, followed by univariate ANOVA and post hoc tests [68,69]. Grouping of dependent variables followed a categorisation in (a) motivational and (b) cognitive outcomes as well as (c) participants’ feedback perception and (d) experienced benefits from feedback, as discussed above. Checking MANOVA assumptions followed the recommendations outlined in Field [70] and specifically included inspection of normality using Shapiro–Wilk tests and covariance homogeneity using Box’s test. Overall, results confirmed that assumptions were sufficiently met. Furthermore, given the roughly equal group sizes and choice of Wilks' Lambda as multivariate test statistic, MANOVA can be assumed to be relatively robust to any occurring moderate violations [70]. Regarding ANOVA if homogeneity of variance was violated, the Games–Howell test was used as a post hoc test; otherwise, Tukey–HSD was used [70]. We report partial η² as effect size, with values of 0.01, 0.06 and 0.14 representing small, medium, and large effects, respectively [72].

## Results

### Usability

Descriptive statistics for the usability variables are presented in the supplementary material in Table S2. Overall, the feedback versions were rated as understandable. Both feedback versions with expert explanations were rated as more comprehensive than the three other feedback versions. For all feedback versions, the participants’ reported that effort and diligence in responding was equivalent and in the upper half of the answer scale. This indicates that they experienced working on the test and feedback tasks as demanding, but the answers for all feedback versions were in a range that did not indicate excessive required effort.

### Comparisons of task-based feedback versions with performance-based feedback (H_1_)

**Figure 1.**
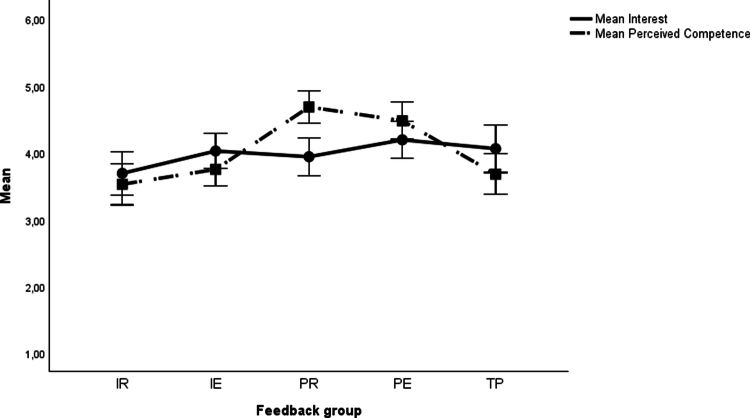
Means and 95% confidence intervals for motivational outcomes. Note: IR = Immediate Reflection, IE = Immediate Explanation, PR = Post-Test Reflection, PE = Post-Test Explanation, TP = Summative Test Profile.

**Figure 2.**
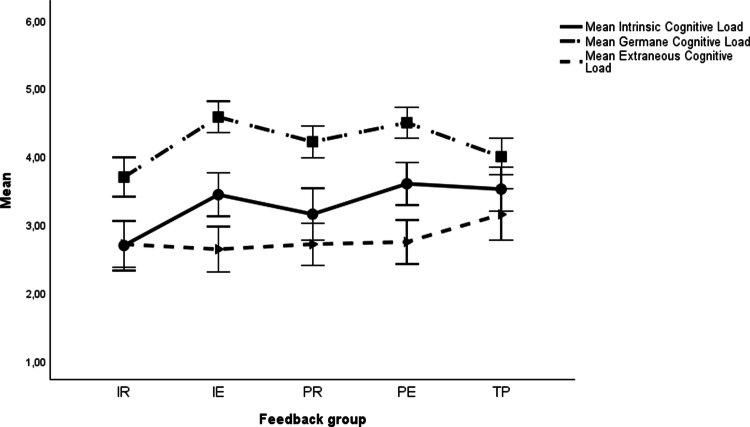
Means and 95% confidence intervals for cognitive load. Note: IR = Immediate Reflection, IE = Immediate Explanation, PR = Post-Test Reflection, PE = Post-Test Explanation, TP = Summative Test Profile.

**Figure 3.**
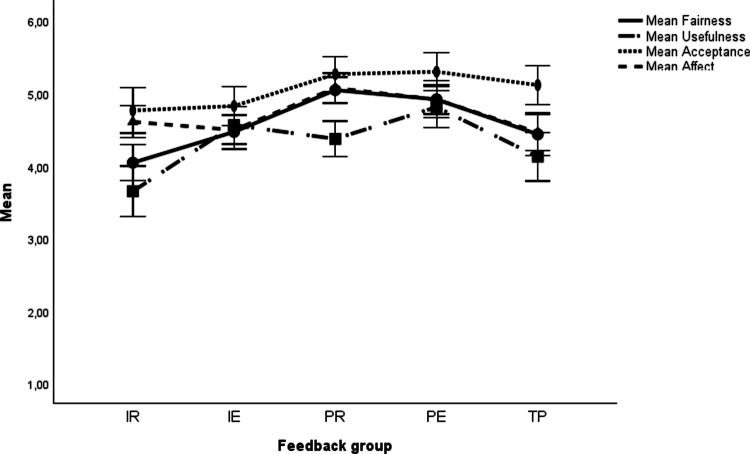
Means and 95% confidence intervals for perception of feedback*.* Note: IR = Immediate Reflection, IE = Immediate Explanation, PR = Post-Test Reflection, PE = Post-Test Explanation, TP = Summative Test Profile.

**Figure 4.**
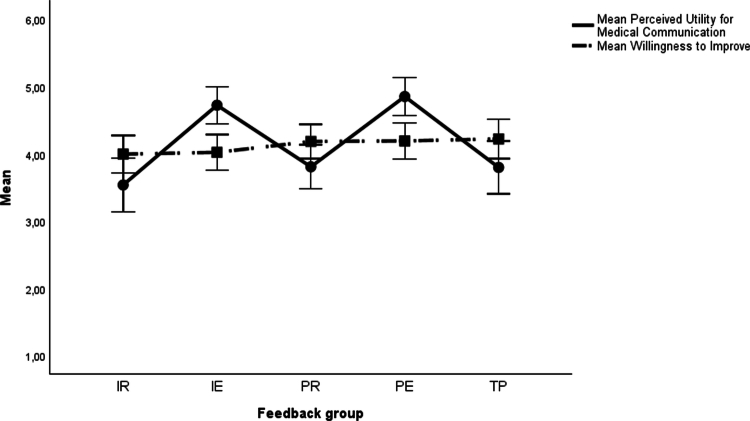
Means and 95% confidence intervals for benefits from feedback. Note: IR = Immediate Reflection, IE = Immediate Explanation, PR = Post-Test Reflection, PE = Post-Test Explanation, TP = Summative Test Profile.

The results for the planned contrast analyses are presented in Table 4 and Figure 1–4. Descriptive statistics are available in the supplementary material (Table S3). For the basic motivational needs, no significant differences were found in *interest* among any of the task-based feedback groups (IR, IE, PR and PE) compared to the performance-based feedback group (TP). Concerning *perceived competence*, there were significant differences in favour of the two feedback groups with delayed feedback (PR and PE) over the TP group, but not for the conditions providing immediate feedback (IR and IE).

**Table 4.** Planned contrasts for task-based feedback groups vs. performance-based feedback group.

Measures	Contrast	Contrast value	*t*	*df*	*p*	Pearson’s *r*
*Basic motivational needs*						
Interest	1	−0.35	−1.61	262	.109	.10
	2	−0.01	−0.07	262	.943	.00
	3	−0.10	−0.47	262	.636	.03
	4	0.15	0.73	262	.467	.04
Perceived competence	1	−0.15	−0.76	261	.451	.05
	2	0.07	0.37	261	.710	.02
	3	1.01	5.14	261	<.001***	.30
	4	0.80	4.14	261	<.001***	.25
*Cognitive Load*						
Intrinsic cognitive load	1	−0.84	−3.45	263	<.001***	.21
	2	−0.10	−0.42	263	.677	.03
	3	−0.37	−1.55	263	.123	.09
	4	0.08	0.34	263	.734	.02
Germane cognitive load	1	−0.31	−1.74	262	.082	.11
	2	0.58	3.38	262	<.001***	.20
	3	0.22	1.26	262	.210	.08
	4	0.50	2.90	262	.004**	.18
Extraneous cognitive load	1	−0.44	−1.81	262	.072	.11
	2	−0.51	−2.21	262	.028^a^	.14
	3	−0.44	−1.86	262	.064	.11
	4	−0.40	−1.74	262	.083	.11
*Perceptions of feedback*						
Fairness	1	−0.39	−2.20	263	.028	.13
	2	0.03	0.20	263	.846	.01
	3	0.61	3.49	263	<.001***	.21
	4	0.48	2.80	263	.006**	.17
Usefulness	1	−0.48	−2.26	263	.025*	.14
	2	0.44	2.13	263	.034*	.13
	3	0.25	1.18	263	.239	.07
	4	0.68	3.33	263	<.001***	.20
Acceptance	1	−0.35	−1.80	263	.074	.11
	2	−0.29	−1.54	263	.125	.09
	3	0.15	0.79	263	.431	.05
	4	0.18	0.98	263	.331	.06
Positive affect	1	0.13	0.82	264	.413	.05
	2	0.02	0.10	264	.918	.01
	3	0.59	3.89	264	<.001***	.23
	4	0.44	2.88	264	.004**	.18
*Benefits from feedback*						
Perceived utility for medical communication	1	−0.28	−1.17	264	.243	.07
	2	0.90	3.90	264	<.001***	.23
	3	−0.13	−0.05	264	.957	.00
	4	1.03	4,41	264	<.001***	.26
Willingness to improve	1	−0.23	−1.14	263	.254	.07
	2	−0.20	−1.05	263	.295	.06
	3	−0.04	−0.19	263	.846	.01
	4	−0.03	−0.16	263	.876	.01

Note: Contrast 1: feedback group IR (Immediate Reflection) against feedback group TP (Summative Test Profile), contrast 2: feedback group IE (Immediate Explanation) against feedback group TP, contrast 3: feedback group PR (Post-Test Reflection) against feedback group TP, contrast 4: feedback group PE (Post-Test Explanation) against feedback group TP. *p < .05. **p < .01. ***p < .001.

a Nonsignificant when corrected for multiple testing using Benjamini-Hochberg correction.

In terms of cognitive load, there was a significant difference in *intrinsic cognitive load* (group IR against TP). In favour of the IR group, the mean value was lower than that of the TP group. There were significant differences in *germane cognitive load*, where the feedback groups with expert explanations (IE and PE) differed significantly from the TP group, favouring the former. None of the other effects on cognitive load were significant.

With respect to the perception of the feedback, there were significant differences concerning *fairness* for the two feedback groups with delayed feedback (PR and PE) and the TP group, favouring the former. For the feedback groups with immediate feedback (IR and IE), there were no significant differences with the TP group. Concerning *usefulness*, there were significant differences in favour of feedback groups with expert explanations (IE and PE) over the TP group, but not for PR. However, there was a significant difference, indicating that the group receiving IR feedback scored lower than the TP group. For *acceptance*, no significant differences occurred. For *positive affect*, the feedback groups with delayed feedback (PR and PE) scored significantly better than the TP group; the groups with immediate feedback (IR and IE) did not differ significantly from the TP group.

As for the benefits of feedback, the *perceived utility for medical communication* was significantly higher in the two feedback groups with expert explanations (IE and PE) than in the TP group. No significant differences occurred between the two feedback groups with reflection prompts (IR and PR) and TP. No significant differences could be found in *willingness to improve*.

In summary, the findings were only partially in line with *H*_1_. Descriptively, this is because performance-based feedback turned out to score relatively high on the dependent variables.

### Comparisons among the task-based feedback versions (H_2_)

For each of the four sets of dependent variables (i.e. basic motivational needs, cognitive load, perceptions of the feedback and benefits from the feedback), the overall MANOVAs were significant (Table S4 in the supplementary material). The results for the follow-up ANOVAs and the corresponding post hoc tests are presented in Table 5. The detailed statistics for each post hoc comparison are provided in Table S5 in the supplementary material.

**Table 5.** One-way analyses of variance and post hoc comparisons for the task-based feedback groups.

Measures	IR	IE	PR	PE	*F* ratio	*p*	Partial η²
*Basic motivational needs*							
Interest^c^	3.694_a_	4.029_a_	3.943_a_	4.196_a_	2.07	.105	.03
Perceived competence^c^	3.531_a_	3.754_a_	4.689_b_	4.482_b_	16.90	<.001***	.19
*Cognitive load*							
Intrinsic cognitive load^c^	2.677_a_	3.421_b_	3.151_a,b_	3.598_b_	5.32	.001**	.07
Germane cognitive load^c^	3.680_a_	4.577_b_	4.214_b_	4.494_b_	10.63	<.001**	.13
Extraneous cognitive load^c^	2.709_a_	2.637_a_	2.711_a_	2.744_a_	0.08	.970	.00
*Perceptions of feedback*							
Fairness^c^	4.047_a_	4.474_a_	5.050_b_	4.923_b_	14.75	<.001***	.17
Usefulness^c^	3.653_a_	4.567_b_	4.377_b_	4.816_b_	12.07	<.001***	.15
Acceptance^c^	4.769_a_	4.830_a_	5.270_a_	5.304_a_	4.35_a,b_	.005**	.06
Positive affect^c^	4.612_a_	4.500_a_	5.079_b_	4.920_a,b_	6.77	<.001***	.10
*Benefits from feedback*							
Perceived utility for medical communication^d^	3.541_a_	4.728_b_	3.811_a_	4.857_b_	16.30	<.001***	.19
Willingness to improve^c^	4.000_a_	4.026_a_	4.189_a_	4.196_a_	0.60	.617	.01

Notes: IR = Immediate Reflection, IE = Immediate Explanation, PR = Post-Test Reflection, PE = Post-Test Explanation. Means with different subscripts differ at the *p *= .05 level by post hoc test. *p < .05. **p < .01. ***p < .001. ^c^Tukey-HSD post hoc test. ^d^Games-Howell post hoc test.

For *interest*, as part of basic motivational needs, there were no significant differences between the four feedback groups (IR, IE, PR and PE). *Perceived competence* was significantly higher in the task-based feedback groups with delayed feedback (PR and PE) than in those receiving immediate feedback (IR and IE). All other effects on motivational needs were nonsignificant.

As far as cognitive load is concerned, the IR group resulted in a significantly lower *intrinsic cognitive load* than both conditions with explanations (IE and PE). For the learning-supportive *germane cognitive load*, the IR group scored significantly lower than the three other three groups (IE, PR and PE). All other differences regarding cognitive load were nonsignificant.

In respect of feedback perception, the participants found *fairness* significantly higher in both delayed feedback conditions (PR and PE) than in the immediate feedback groups (IR and IE). Moreover, the IR group was perceived as significantly less generally *useful* than the other three groups (IE, PR and PE). The same pattern of results occurred for *positive affect*. For *acceptance*, there were significantly lower mean values for the IR group compared to the PE group. All other effects on feedback perception were nonsignificant.

Finally, regarding the benefits from the feedback, *perceived utility for medical communication* was significantly higher in the groups with expert explanations (IE and PE) than in the groups with reflection prompts (IR and PR). All other effects on benefits from the feedback were nonsignificant.

In summary, the results on *H*_2_ were mostly in line with the expectations. The overall MANOVAs confirmed the differential effects of the different feedback versions on the outcome variables. However, we did not observe statistically significant group differences in all investigated outcomes.

## Discussion

This study examined how automated feedback can be effectively integrated into an online SJT for the formative assessment of medical communication competence in medical education [4]. We developed four task-based feedback versions that differed in feedback enrichment and timing, as well as one performance-based feedback version, and evaluated their effects on motivational and cognitive factors, feedback perception and perceived benefits—key influences on students’ ongoing learning processes after feedback.

Regarding *H*_*1*_, one possible explanation for the unexpectedly favourable results of performance-based feedback on some outcome variables is that medical students may be more receptive to this type of feedback than other target groups [10]. They are accustomed to test-based assessment formats [73] and often perceive these assessments primarily as measures of their current knowledge and competence rather than as learning opportunities [74]. This aligns with research indicating that competitiveness plays a significant role in medical education [75,76] and that medical students respond positively to competitive learning environments [76,77]. Performance-based feedback may therefore have resonated with their familiar learning strategies. Similarly, test score profiles incorporating social comparisons may introduce elements of gamification [78], which can further enhance engagement and motivation. Nevertheless, despite these positive findings, performance-based feedback has inherent limitations, as it primarily conveys information about current performance without offering explicit directions on how to improve [4,10,46].

Regarding *H*_2_, our results align with previous research indicating that different feedback types are differentially effective and that no single approach is clearly superior [10,23]. Accordingly, a differentiated pattern emerged across outcomes, underscoring the importance of aligning feedback design with specific learning objectives.

### Discussion of results by outcome variable

All five feedback versions were perceived as equally *interesting*, suggesting a generally engaging feedback experience that supports intrinsic motivation for further learning. *Perceived competence*, an important motivational factor, was particularly enhanced by the delayed feedback versions (PR and PE), indicating that feedback provided after test completion improves satisfaction and reinforces a sense of competence.

For *intrinsic cognitive load*, version IR exhibited the lowest values, likely due to its simplicity and lack of added information [65]. While intrinsic cognitive load was somewhat higher for the other task-based feedback versions, it remained moderate overall, indicating that the feedback did not impose excessive cognitive demands. Regarding *germane cognitive load*, which reflects elaboration processes conducive to learning, the expert explanation versions (IE and PE) yielded the highest values, underscoring their potential to support deeper understanding. Students in these conditions were also more motivated to ensure correct comprehension [65]. Hence, from a cognitive perspective, these conditions appear most conducive to fostering learning after feedback. No significant differences in *extraneous cognitive load* emerged. Descriptively performance-based feedback (TP) yielded the highest value, possibly because it did not provide feedback linked to the content of the tasks.

All feedback versions were well accepted by participants, corroborating earlier findings that students value feedback in online SJTs [19]. Task-based feedback provided after test completion (PR and PE) was perceived as *fairer* and most strongly supported *positive affect*. Regarding perceived *usefulness*, the IR version was rated as least informative, potentially offsetting its advantages in terms of cognitive load. Feedback enriched with expert explanations (IE and PE) was perceived as most useful, both overall and for learning medical communication, suggesting that it effectively provides relevant guidance for improvement. This perception aligns with the enhanced learning potential from a cognitive load perspective. Notably, *willingness to improve* was descriptively highest in the performance-based group, although this effect was nonsignificant and should be interpreted cautiously given the low reliability of the scale (Table 3). Potentially, the summative test profile highlights strengths and weaknesses in a way that triggers motivation to improve future performance.

### Discussion of results by enrichment strategy

As for broader implications for feedback enrichment strategies, our findings on *reflection prompts* support research indicating that engagement in reflective activities hinges on perceived task relevance and feasibility [79]. Given their stage of study, most participants should have had at least some basic knowledge of medical communication; however, students with more limited understanding may have found it difficult to engage meaningfully in reflection. This suggests that reflection-based feedback may be more effective after initial training in medical communication. Moreover, brief preparatory training in reflective writing or the use of reflection prompts (e.g. in form of brief guiding questions [80]) may improve engagement with such tasks in practical applications [81]. Despite the introductory screencast presented in the experiment, some participants may have been unsure how to reflect, which might partly explain the outcomes in the IR and PR conditions. While reflection training was deliberately omitted for the methodological reasons discussed above, future research should examine how structured reflection guidance interacts with feedback timing and enrichment strategies in SJT-based learning contexts.

Findings regarding *expert explanations* support prior evidence that feedback is particularly helpful when it is detailed and informative [41]. However, some caveats are in order. First, there is a trade-off between informational richness and text length. One possible solution is to present brief explanations initially while offering optional access to more detailed information. Second, what supports learning is not always optimal for motivation: in the present study, the IE condition led to somewhat reduced motivational and affective outcomes, whereas PE appeared to offer a more balanced trade-off.

With respect to feedback timing, our results indicate a preference for *post-test feedback*. While immediate feedback allows real-time strategy adjustment, delayed feedback enables uninterrupted task completion and may reduce distraction [48]. This advantage may partly reflect medical students’ familiarity with assessment formats in which feedback is provided after test completion [82]. Delayed feedback may therefore facilitate a more comprehensive performance analysis.

### Limitations and future research

Several limitations should be acknowledged. First, selection bias cannot be ruled out, as students with higher motivation or interest in medical communication may have been more likely to participate. Although monetary incentives were used to mitigate this risk and randomised assignment ensured internal validity, generalisability may be limited. Second, the study does not provide direct insights into learning processes and how the feedback actually fostered participants’ learning. This is a common challenge in research on self-regulated learning, where tracking such processes is inherently difficult [83]. Third, despite the detailed instructions and a preparatory screencast, we lack behavioural data beyond self-reports to verify whether students used the feedback as intended. Future longitudinal research could address this using learning analytics methods, such as log file analysis [84] or learning journals, to track and analyse participants’ interactions with feedback, monitor learning progress, and examine lasting effects. Such methods could not be implemented in the present study because data protection regulations prohibited the collection of learning analytics via the learning platform. Fourth, we cannot determine whether results would differ in a longer learning session with more test tasks. Using the full test was infeasible due to time constraints. This challenge is common in formative testing when students must process feedback in addition to completing test tasks. For practical applications, this underscores the importance of carefully selecting a limited number of test tasks aligned with the key learning objectives [85].

## Conclusion

This study demonstrated the integration of automated formative and summative feedback into an online SJT in medical education. The findings show that feedback design choices differentially influence factors shaping students’ feedback processing and subsequent learning. Selecting an appropriate feedback design therefore involves balancing competing objectives, such as motivation or cognitive load, while aligning feedback with the intended learning goals and application context.

## Supplementary Material

10_Reiser_etal_Supplementary material.docx 10_Reiser_etal_Supplementary material.docx

## Data Availability

The VA-MeCo’s test materials are available in the OpenTestArchive repository [86]. The dataset (including the codebook) generated and/or analysed during the current study is available in the PsychArchives data repository [67].

## Endnotes

1 Here, we use the terms ʻformative assessment’ and ʻassessment for learning’ synonymously, while acknowledging that conceptual distinctions between the two exist [28].

2 Detailed information on the VA-MeCo’s test development and design is available in [4].
