# Supplementary material for: Integrating formative and summative feedback in online situational judgement tests: effects of feedback design on medical students’ motivational and cognitive learning factors
Source: Med Educ Online. 2026 Mar 2;31(1):2639198. doi: 10.1080/10872981.2026.2639198 (PMC12973788; doi:10.1080/10872981.2026.2639198)
Supplement: 10_Reiser_etal_Supplementary material.docx [file ZMEO_A_2639198_SM4149.docx]

**Supplementary material**

**Integrating Formative and Summative Feedback in Online Situational Judgement Tests: Effects of Feedback-Design on Medical Students' Motivational and Cognitive Learning Factors**

**Table S1**

*Contrast codes for planned contrasts comparing task-based feedback groups vs. performance-based group*

| Contrasts | Codes for Feedback Groups | | | | |
| --- | --- | --- | --- | --- | --- |
|  | IR | IE | PR | PE | TP |
| 1 | 1 | 0 | 0 | 0 | -1 |
| 2 | 0 | 1 | 0 | 0 | -1 |
| 3 | 0 | 0 | 1 | 0 | -1 |
| 4 | 0 | 0 | 0 | 1 | -1 |

*Note.* IR = Immediate Reflection, IE = Immediate Explanation, PR = Post-Test Reflection, PE = Post-Test Explanation, TP = Summative Test Profile.

**Table S2**

*Descriptive statistics for usability*

| Measures | IR | | IE | | PR | | PE | | TP | |
| --- | --- | --- | --- | --- | --- | --- | --- | --- | --- | --- |
|  | *n* | *M (SD)* | *n* | *M (SD)* | *n* | *M (SD)* | *n* | *M (SD)* | *n* | *M (SD)* |
| Clarity of the feedback^a^ | 49 | 3.59 (1.25) | 57 | 4.80 (0.98) | 53 | 4.00 (1.02) | 56 | 4.97 (0.91) | 54 | 3.83 (1.28) |
| Feedback comprehensiveness^b^ | 49 | 2.53 (1.06) | 57 | 4.46 (0.89) | 53 | 2.81 (1.14) | 56 | 4.46 (0.99) | 54 | 3.46 (1.14) |
| Diligence^a^ | 49 | 3.94 (1.13) | 57 | 4.03 (1.07) | 53 | 3.99 (1.23) | 56 | 3.63 (0.95) | 54 | 3.91 (1.35) |
| Effort^c^ | 49 | 4.51 (1.24) | 57 | 4.08 (1.36) | 53 | 3.38 (1.33) | 56 | 3.54 (1.29) | -^d^ | -^d^ |

*Note.* IR = Immediate Reflection, IE = Immediate Explanation, PR = Post-Test Reflection, PE = Post-Test Explanation, TP = Summative Test Profile.

^a^ 6-point rating scale (1 = *strongly disagree*, 6 = *strongly agree*, two items).

^b^ 6-point rating scale (1 = *too low*, 6 = *too high*, 1 item).

^c^ 6-point rating scale (1 = *strongly disagree*, 6 = *strongly agree*, two items, adapted for each feedback version); recoded values so that higher values stand for less effort.

^d^ Effort was not measured for group TP.

**Table S3**

*Descriptive statistics of the outcome variables*

| Measures | IR | | IE | | PR | | PE | | TP | |
| --- | --- | --- | --- | --- | --- | --- | --- | --- | --- | --- |
|  | *n* | *M (SD)* | *n* | *M (SD)* | *n* | *M (SD)* | *n* | *M (SD)* | *n* | *M (SD)* |
| *Basic motivational needs* |  |  |  |  |  |  |  |  |  |  |
| Interest | 48 | 3.69 (1.12) | 57 | 4.03 (0.99) | 53 | 3.94 (1.03) | 56 | 4.20 (1.03) | 53 | 4.04 (1.27) |
| Perceived  competence | 48 | 3.53 (1.07) | 57 | 3.75 (0.94) | 53 | 4.69 (0.88) | 56 | 4.48 (1.04) | 52 | 3.68 (1.09) |
| *Cognitive load* |  |  |  |  |  |  |  |  |  |  |
| Intrinsic cognitive  load | 48 | 2.68 (1.24) | 57 | 3.42 (1.18) | 53 | 3.15 (1.38) | 56 | 3.60 (1.17) | 54 | 3.52 (1.17) |
| Germane cognitive  load | 48 | 3.68 (0.98) | 56 | 4.58 (0.87) | 53 | 4.21 (0.85) | 56 | 4.49 (0.85) | 54 | 3.99 (0.99) |
| Extraneous cognitive  load | 47 | 2.71 (1.14) | 57 | 2.64 (1.23) | 53 | 2.71 (1.12) | 56 | 2.74 (1.20) | 54 | 3.15 (1.38) |
| *Perceptions of feedback* |  |  |  |  |  |  |  |  |  |  |
| Fairness | 49 | 4.05 (0.86) | 57 | 4.47 (0.90) | 53 | 5.05 (0.66) | 56 | 4.92 (0.95) | 53 | 4.44 (1.07) |
| Usefulness | 49 | 3.65 (1.22) | 57 | 4.57 (0.96) | 53 | 4.38 (0.88) | 56 | 4.82 (1.05) | 53 | 4.13 (1.22) |
| Acceptance | 49 | 4.77 (1.10) | 57 | 4.83 (1.02) | 53 | 5.27 (0.87) | 56 | 5.30 (0.97) | 53 | 5.12 (0.97) |
| Positive affect | 49 | 4.61 (0.77) | 57 | 4.50 (0.76) | 53 | 5.08 (0.75) | 56 | 4.92 (0.75) | 54 | 4.48 (0.90) |
| *Benefits from feedback* |  |  |  |  |  |  |  |  |  |  |
| Perceived utility for  medical  communication | 49 | 3.54 (1.38) | 57 | 4.73 (1,04) | 53 | 3.81 (1.20) | 56 | 4.86 (1.07) | 54 | 3.82 (1.43) |
| Willingness to  improve | 49 | 4.00 (0.97) | 57 | 4.03 (1.01) | 53 | 4.20 (0.93) | 56 | 4.20 (1.02) | 53 | 4.23 (1.06) |

*Note.* IR = Immediate Reflection, IE = Immediate Explanation, PR = Post-Test Reflection, PE = Post-Test Explanation, TP = Summative Test Profile.

**Table S4**

*Multivariate analysis of variance for four task-based feedback versions*

| Measures | *Wilks’s Λ* | *F* ratio | *df* | Partial η² |
| --- | --- | --- | --- | --- |
| Basic motivational needs | .79 | 8.77*** | 6,418 | .11 |
| Cognitive load | .84 | 4.09*** | 9,501 | .06 |
| Perceptions of feedback | .71 | 6.49*** | 12,550 | .11 |
| Benefits from feedback | .79 | 8.61*** | 6,420 | .11 |

*Note. Λ =* Wilks’s Lambda.

*** *p* < .001.

**Table S5**

*Post hoc comparisons of the four task-based feedback versions*

| Measures | Comparisons | Mean Difference | *SE* | *p* | 95% CI |
| --- | --- | --- | --- | --- | --- |
| *Basic motivational needs* |  |  |  |  |  |
| Interest^a^ | IR vs. IE | -0.33 | 0.20 | .359 | [-0.9, 0.2] |
|  | IR vs. PR | -0.25 | 0.21 | .628 | [-0.8, 0.3] |
|  | IR vs. PE | -0.50 | 0.21 | .071 | [-1.0, 0.0] |
|  | IE vs. PR | 0.09 | 0.20 | .973 | [-0.4, 0.6] |
|  | IE vs. PE | -0.17 | 0.20 | .829 | [-0.7, 0.3] |
|  | PR vs. PE | -0.25 | 0.20 | .585 | [-0.8, 0.3] |
| Perceived competence^a^ |  |  |  |  |  |
|  | IR vs. IE | -0.22 | 0.19 | .652 | [-0.7, 0.3] |
|  | IR vs. PR | -1.12 | 0.20 | < .001*** | [-1.7, -0.7] |
|  | IR vs PE | -0.95 | 0.20 | < .001*** | [-1.5, -0.5] |
|  | IE vs. PR | -0.93 | 0.19 | < .001*** | [-1.4, -0.4] |
|  | IE vs. PE | -0.73 | 0.18 | < .001*** | [-1.2, -0.3] |
|  | PR vs. PE | 0.21 | 0.19 | 0.690 | [-0.3, 0.7] |
| *Cognitive load* |  |  |  |  |  |
| Intrinsic cognitive load^a^ | IR vs. IE | -0.74 | 0.24 | .013* | [-1.4, -0.1] |
|  | IR vs. PR | -0.47 | 0.25 | .226 | [-1.1, 0.2] |
|  | IR vs. PE | -0.92 | 0.24 | .001** | [-1.6, -0.3] |
|  | IE vs PR | 0.27 | 0.24 | 0.666 | [-0.3, 0.9] |
|  | IE vs. PE | -0.18 | 0.23 | 0.873 | [-0.8, 0.4] |
|  | PR vs. PE | -0.45 | 0.24 | 0.241 | [-1.1, 0.2] |
| Germane cognitive load^a^ | IR vs. IE | -0.90 | 0.17 | < .001*** | [-1.3, -0.4] |
|  | IR vs. PR | -0.53 | 0.18 | .015* | [-1.0, -0.1] |
|  | IR vs. PE | -0.81 | 0.17 | < .001 | [-1.3, -0.4] |
|  | IE vs. PR | 0.36 | 0.17 | .142 | [-0.1, 0.8] |
|  | IE vs. PE | 0.08 | 0.17 | .959 | [-0.3, 0.5] |
|  | PR vs. PE | -0.28 | 0.17 | .351 | [-0.7, 0.2] |
| Extraneous cognitive load^a^ | IR vs. IE | 0.07 | 0.23 | .990 | [-0.5, 0.7] |
|  | IR vs. PR | 0.00 | 0.23 | 1.000 | [-0.6, 0.6] |
|  | IR vs. PE | -0.3 | 0.23 | .999 | [-0.6, 0.6] |
|  | IE vs. PR | -0.07 | 0.22 | .988 | [-0.7, 0.5] |
|  | IE vs. PE | -0.11 | 0.22 | .963 | [-0.7, 0.5] |
|  | PR vs. PE | -0.03 | 0.23 | .999 | [-0.6, 0.5] |
| *Perceptions of feedback* |  |  |  |  |  |
| Fairness^a^ | IR vs. IE | -0.43 | 0.17 | 0.053 | [-0.9, 0.0] |
|  | IR vs. PR | -1.00 | 0.17 | < .001*** | [-1.4, -0.6] |
|  | IR vs. PE | -0.88 | 0.17 | < .001*** | [-1.3, -0.4] |
|  | IE vs. PR | -0.58 | 0.16 | .003** | [-1.0, -0.2] |
|  | IE vs. PE | -0.45 | 0.16 | .028* | [-0.9, 0.0] |
|  | PR vs. PE | 0.13 | 0.16 | .863 | [-0.3, 0.6] |
| Usefulness^a^ | IR vs. IE | -0.91 | 0.20 | < .001*** | [-1.4, -0.4] |
|  | IR vs. PR | -0.72 | 0.20 | .003** | [-1.3, -0.2] |
|  | IR vs. PE | -1.16 | 0.20 | < .001*** | [-1.7, -0.6] |
|  | IE vs. PR | 0.19 | 0.20 | .770 | [-0.3, 0.7] |
|  | IE vs. PE | -0.25 | 0.19 | .577 | [-0.8, 0.3] |
|  | PR vs. PE | -0.44 | 0.20 | .122 | [-0.9, 0.1] |
| Acceptance^a^ | IR vs. IE | -0.06 | 0.19 | .989 | [-0.6, 0.4] |
|  | IR vs. PR | -0.50 | 0.20 | .055 | [-1.0, 0.0] |
|  | IR vs. PE | -0.53 | 0.19 | .032 | [-1.0, 0.0] |
|  | IE vs. PR | -0.44 | 0.19 | .095 | [-0.9, 0.0] |
|  | IE vs. PE | -0.47 | 0.19 | .057 | [-1.0, 0.0] |
|  | PR vs. PE | -0.03 | 0.19 | .998 | [-0.5, 0.5] |
| Positive affect^a^ | IR vs. IE | 0.11 | 0.15 | .872 | [-0.3, 0.5] |
|  | IR vs. PR | -0.47 | 0.15 | .012* | [-0.9, -0.1] |
|  | IR vs. PE | -0.31 | 0.15 | .165 | [-0.7, 0.1] |
|  | IE vs. PR | -0.60 | 0.14 | < .001*** | [-1.0, -0.2] |
|  | IE vs. PE | -0.42 | 0.14 | .019* | [-0.8, -0.1] |
|  | PR vs. PE | 0.16 | 0.15 | .693 | [-0.2, 0.5] |
| *Benefits from feedback* |  |  |  |  |  |
| Perceived utility for medical  communication^b^ | IR vs. IE | -1.19 | 0.24 | < .001*** | [-1.8, -0.6] |
|  | IR vs. PR | -0.27 | 0.26 | .719 | [-0.9, 0.4] |
|  | IR vs. PE | -1.32 | 0.24 | < .001*** | [-2.0, -0.7] |
|  | IE vs. PR | 0.92 | 0.21 | < .001*** | [0.4, 1.5] |
|  | IE vs. PE | -0.13 | 0.20 | .915 | [-0.6, 0.4] |
|  | PR vs. PE | -1.05 | 0.22 | < .001*** | [-1.6, -0.5] |
| Willingness to improve^a^ | IR vs. IE | -0.03 | 0.19 | .999 | [-0.5, 0.5] |
|  | IR vs. PR | -0.19 | 0.19 | .767 | [-0.7, 0.3] |
|  | IR vs. PE | -0.20 | 0.19 | .737 | [-0.7, 0.3] |
|  | IE vs. PR | -0.16 | 0.19 | .827 | [-0.6, 0.3] |
|  | IE vs. PE | -0.17 | 0.18 | .794 | [-0.6, 0.3] |
|  | PR vs. PE | -0.01 | 0.19 | 1.000 | [-0.5, 0.5] |

*Note.* IR = Immediate Reflection, IE = Immediate Explanation, PR = Post-Test Reflection, PE = Post-Test Explanation.

**p* < .05. ***p* < .01. ****p* < .001.

^a^ Tukey-HSD was used as post hoc test.

^b^ Games-Howell was used as post hoc test.
